# Unraveling the genomic determinants of response to CDK4/6 inhibitors

**DOI:** 10.18632/oncotarget.26109

**Published:** 2018-12-07

**Authors:** Lacey M. Litchfield, Vue W. Webster, Sean G. Buchanan

**Affiliations:** Sean G. Buchanan: Eli Lilly and Company, Indianapolis, IN, USA

**Keywords:** CDK4/6, CDK2, CCND1, CCND2, CCND3

Aberrant activation of the D-cyclin-CDK4/6-Rb pathway and subsequent G1/S cell cycle progression is a hallmark of many cancers. Targeting this axis via the CDK4/6 inhibitor drug class is revolutionizing the treatment of hormone receptor-positive breast cancer, where cyclin D1 expression driven by ER signaling may explain the excellent clinical activity of these drugs. Beyond breast cancer, many other cancers subvert the Rb pathway through various mechanisms unleashing D-cyclin-CDK4/6 activity, including cancers with mitogenic signaling dysregulation (RTKs, Ras, PI3K), expression of oncogenic transcription factors (TFs; Myc, β-catenin, AR, EWS-FLI), loss of the negative regulator of CDK4/6 p16^INK4A^/*CDKN2A*, or genomic alterations in the D-cyclin genes themselves (mutation, translocation, or amplification) [1, 2]. These additional subsets of cancers might also be expected to depend on CDK4/6 activity and, thus, be sensitive to CDK4/6 inhibitors.

We recently compared the relative sensitivities of a panel of genomically characterized cell lines to the CDK4/6 inhibitor abemaciclib and found that, while some of these expectations were met, including increased sensitivity of cells with D-cyclin gene translocation or high expression of *CCND1-3* driven by oncogenic transcription factors (e.g., Ewing's sarcoma, medulloblastoma, neuroblastoma), cells with certain other mutations that might be expected to increase sensitivity to CDK4/6 inhibition were, surprisingly, relatively insensitive at clinical concentrations (e.g., *CCND1* amplification in esophageal cancer, *KRAS* mutation, *CDKN2A* loss) [3]. Mechanistically, we observed that maintenance of CDK2 activity in *KRAS/CDKN2A* mutant cancers allows these cells to overcome the growth-suppressive effects of abemaciclib.

We also described a particularly sensitive subset of cancers with D-cyclin activating features (DCAF), a composite class of genetic alterations that includes *CCND1* t(11;14) translocation, *CCND1-3* 3'UTR loss, *CCND2* or *CCND3* amplification, K-cyclin (KSHV), and *FBXO31* loss [3], features found overall in ~10% of the cancers in TCGA. The association of these genetic features with sensitivity to CDK4/6 inhibition allows for the identification of additional tumor types that may benefit from CDK4/6i, such as uveal melanoma *(CCND3* amplification 23%) and testicular germ cell tumors *(CCND2* amplification 67%) (Table 1), the latter of which have previously shown a positive response to palbociclib clinically [4].

**Table 1:** TCGA tumor samples with high frequency of D-cyclin activating features (DCAF) based on 11426 TCGA patient tumor samples across 37 tumor types t(11;14) frequencies in MCL and myeloma are taken from a survey of the literature

Tumor Type	DCAF 0/o	Notes
Mantle cell lymphoma	95%+	t(11;14); ~20% *CCND1* 3'UTR loss may represent highly sensitive subset
Testicular germ cell tumors	67%	67% *CCND2* amp
Uterine carcinosarcoma	32%	16% *CCND2;* 19% *CCND3* amp
Ovarian serous cystadenocarcinoma	30%	20% *CCND2* amp, 9% *CCND3* amp
Myeloma	10-30%	t(11;14); also some 3'UTR loss
Uveal melanoma	25%	23% *CCND3* amp
Adrenocortical carcinoma	20%	18% *CCND2* amp
DLBCL	20%	*CCND2* 3'UTR loss
Melanoma	15%	11% *CCND3* amp
Gastric cancer	14%	6% *CCND3* amp, 6% 3'UTR loss
Esophageal cancer	13%	8% *CCND3* amp
Uterine corpus endometrial carcmoma	13%	7% *CCND1* 3'UTR loss
Lung squamous	11%	8% *CCND2* amp
Sarcoma	11%	6% *CCND2* amp, 5% *CCND3* amp
Head and neck squamous	10%	8% *CCND2* amp
Rectal cancer	10%	3% *CCND2* amp, 3% *CCND3* amp

Another of the identified DCAF alterations that merits further investigation is *CCND1-3* 3'UTR loss. Previously characterized in mantle cell lymphoma (MCL), loss of *CCND1* 3'UTR is associated with increased *CCND1* mRNA stability and poor clinical prognosis [5]. While abemaciclib has displayed monotherapy activity in unselected relapsed or refractory MCL [6], it would be interesting to test the subset of patients with *CCND1* 3'UTR loss on the basis of these findings (potentially in combination with BTK or PI3Kδ inhibitors). Beyond MCL, 3'UTR loss also occurs in additional tumor types that may be pursued clinically, including our identification of novel mutations resulting in 3'UTR loss in endometrial cancer [3].

Overall, this study begins to reveal the rules for sensitivity to CDK4/6 inhibitors. Expression of Rb, the primary target of CDK4/6, is required for a high level of sensitivity to CDK4/6 inhibition, but many Rb+ tumors fail to respond to treatment at clinical concentrations. The finding that *CCNE1* amplification is associated with resistance suggests some cancers can compensate for CDK4/6 inhibition with CDK2, an association further supported by the maintenance of CDK2 activity in other cells of intermediate sensitivity (e.g, *KRAS, CDKN2A* mutant cells) [3]. Consistent with these findings, high *CCNE1* expression was associated with reduced benefit from palbociclib clinically [7] and *CCNE1* amplification has also been observed in acquired resistance to CDK4/6 inhibitors in *vitro* [8]. The strong association of *TP53* mutation with resistance could also be indicative of another path to CDK2 activation, since *TP53* mutation should diminish p21 levels and permit a CDK2 bypass of CDK4/6.

**Figure 1:**
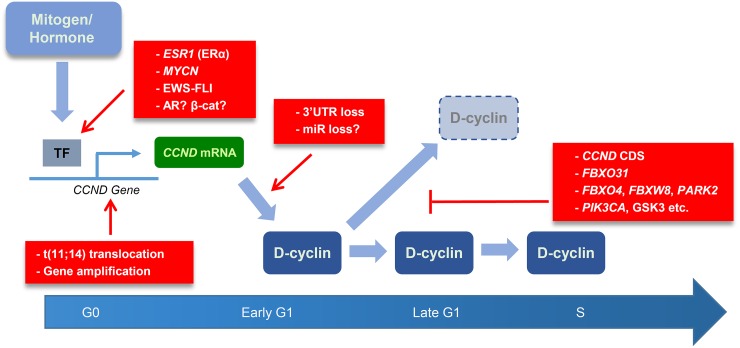
Two-hit model for D-cyclin-driven CDK4/6i sensitivity

It is intriguing that many of the most sensitive cell lines have multiple features expected to dysregulate D-cyclins, including uncoupling transcription from normal strict control (e.g., *CCND* amplification, t(11;14) translocation; ER, AR, Myc TFs) and enhancing protein half-life either by increasing translation (mRNA stability with 3'UTR loss, miRs, PI3K/mTOR) or reducing protein turnover (point mutations, *FBXO4, FBXW8, PARK2, FBXO31*). While gross D-cyclin mRNA and protein levels in unsynchronized cells did not clearly correlate with abemaciclib response, increasing the half-life of D-cyclin mRNA and protein would be expected to prolong the production of an active D-cyclin/CDK complex through the G1 phase into S, rendering the cells self-sufficient with D-cyclin kinases to drive the cell cycle (Figure 1). Also interesting in this regard is that the MCL subset with 3'UTR loss and poor prognosis have dramatically stabilized mRNA but only modest increases in mRNA and protein expression in unsynchronized cells [5].

In summary, our findings suggest that the combination of (a) *RB1/CCNE/TP53* wt status and (b) mutations that hit two steps in D-cyclin production (transcription and protein half-life) might be expected to signify abemaciclib monotherapy benefit. For other Rb+ tumors (e.g., *KRAS/CDKN2A* mutant, *CCNE* amplified), combination strategies that neutralize the CDK2 bypass may prove to be effective.
